# Large‐scale phenotyping of physical and antioxidant traits in peach and apricot cultivars

**DOI:** 10.1002/jsfa.70661

**Published:** 2026-04-16

**Authors:** Pavlina Drogoudi, Georgios Pantelidis, Elli Diakaki, Konstantina Ziakou, Dimitrios Gerasopoulos

**Affiliations:** ^1^ Department of Deciduous Fruit Trees Institute of Plant Breeding and Genetic Resources, Hellenic Agricultural Organization – DIMITRA Naoussa Greece; ^2^ Laboratory of Food Engineering and Processing, Department of Food Science & Technology Faculty of Agriculture, Aristotle University of Thessaloniki Thessaloniki Greece

**Keywords:** antioxidants, carotenoids, fruit color, *Prunus armeniaca* L., *Prunus persica* L. Batsch, total phenolics

## Abstract

**BACKGROUND:**

Peach and apricot are valued for their sensory attributes and nutritional value; however large‐scale evaluations of fruit quality and antioxidant‐related traits remain limited. This study aimed to quantify phenotypic diversity and examine relationships between fruit quality and antioxidant traits in 100 peach and 32 apricot cultivars grown under the same experimental conditions.

**RESULTS:**

Substantial phenotypic diversity was observed for fruit quality and antioxidant traits. Total phenolic contents showed the greatest variation, differing by up to approximately sevenfold among peach and apricot cultivars, while high‐antioxidant genotypes were relatively rare. Commercially important cultivars with high antioxidant contents were identified, highlighting potential value for health‐oriented markets; of the most widely used cultivars it was ‘Andross’ and ‘Big Top’ that may be considered for having high nutritional importance to consumers. Ripening date was only correlated with antioxidant content in nectarine and apricot, indicating that it should not be regarded as a universal predictor of antioxidant accumulation. Juiciness, an important quality trait, varied nearly twofold in both species. Associations among peel and flesh color traits differed among fruit types. Multivariate analyses distinguished cultivar groups, with French‐origin apricot cultivars associated with higher antioxidant content, later ripening, and higher ripening index, whereas Greek‐origin cultivars clustered separately with generally lower antioxidant levels and reduced peel coloration.

**CONCLUSION:**

The observed diversity provides valuable opportunities for selecting cultivars with desirable sensory attributes and high nutritional value, thereby supporting the development of targeted fruit ideotypes and providing guidance for nutritionally aware consumers. © 2026 The Author(s). *Journal of the Science of Food and Agriculture* published by John Wiley & Sons Ltd on behalf of Society of Chemical Industry.

## INTRODUCTION

Peach (*Prunus persica* L. Batsch) and apricot (*Prunus armeniaca* L.) are among the most economically significant temperate fruit species – ranked third and sixth globally in production volume, respectively, with peach following apple and pear, and apricot following peach and plum – valued both for fresh consumption and for use in processed products such as juices, jams, and canned fruits.^1^ They are widely appreciated for their attractive appearance, pleasant flavor, and nutritional value. Beyond their sensory attributes, these fruits are recognized as valuable sources of bioactive compounds, including phenolic compounds and carotenoids, which contribute to antioxidant capacity and are associated with potential health benefits related to the reduction of oxidative stress.^2^, ^3^

Peach and apricot cultivars produce fruit that display considerable diversity in ripening date (RD) and morphological and physicochemical traits, which underpin their classification and suitability for various end‐uses. Based on fruit shape, peach cultivars are broadly categorized as either round (conventional) or flat (FLAT). In addition to shape, peach flesh color (yellow or white) and peel pigmentation (red‐blushed or pale) are key attributes governed by the presence or absence of anthocyanin and carotenoid pigments. Flesh texture is another critical trait for peach and forms the basis for the distinction between melting (MPE) and nonmelting (NMPE) types. MPE‐flesh peaches undergo rapid softening during ripening and are thus favored for fresh‐market consumption, whereas NMPE‐flesh peaches retain firmness upon ripening, a trait makes them suited for industrial processing and canning.^4^

Consumer acceptance of peach and apricot fruits is primarily driven by physical and chemical quality traits such as fruit size, peel and flesh color, juiciness, soluble solids content (SSC), titratable acidity (TA), and their balance expressed as ripening index (RI).^5^, ^6^, ^7^ Recent findings indicate that preharvest conditions (e.g. cultivar genetics, orchard management, and environmental factors) exert a greater influence on SSC than postharvest factors, highlighting their predominant role in shaping this key quality parameter.^5^

In parallel, growing consumer awareness of the link between diet and health has increased interest in fruits with enhanced nutritional and functional properties, particularly those with high antioxidant content. In stone fruits, phenolic compounds represent the major contributors to antioxidant capacity, while carotenoids, especially abundant in apricot fruit, also contribute to nutritional value and visual quality.^3^, ^8^, ^9^

In recent decades, the number of commercially available peach and apricot cultivars has expanded significantly due to advances in breeding programs, producing cultivars varying widely in appearance, ripening time, taste, and postharvest performance. While this genetic and phenotypic diversity represents a valuable opportunity for precision orchard planning, it has also introduced considerable complexity for growers due to inconsistent cultivar performance across environments that often lead to confusion in selecting the most suitable cultivars for sustained productivity and profitability. As a result, there is a growing need for region‐specific cultivar evaluation trials and decision‐support tools to assist growers in optimizing their planting choices.^10^, ^11^, ^12^ Previous studies have reported substantial variability in physical, chemical, and antioxidant‐related traits among peach and apricot cultivars.^11^, ^13^, ^14^ However, many investigations have focused on a limited number of genotypes, which limits the ability to draw general conclusions applicable to breeding and cultivar selection. Moreover, integrated evaluations that simultaneously consider fruit quality attributes and antioxidant traits across different peach types (MPE, NMPE, nectarine (NE), FLAT) and apricot cultivars under uniform environmental conditions remain limited.^10^, ^12^, ^14^, ^15^

The objective of the study presented here was to conduct a comprehensive, large‐scale evaluation of physical and functional traits in a diverse set of peach and apricot cultivars grown under the same environmental conditions. By integrating conventional fruit quality measurements with assessments of phenolic content and antioxidant capacity, the work aimed to (i) quantify phenotypic diversity within and between species, (ii) elucidate relationships among fruit quality and antioxidant traits, and (iii) identify cultivar profiles of interest for breeding programs targeting improved physical, sensory, and nutritional fruit quality. Owing to logistical constraints associated with the management and evaluation of a large number of genotypes, measurements for the two species were conducted in different years. As a result, interspecific comparisons should be interpreted with caution, as year‐to‐year climatic variation may have influenced the observed phenotypic responses.^16^, ^17^

## MATERIALS AND METHODS

### Plant material and climatic parameters

The study included 100 peach and 32 apricot cultivars originating from international breeding programs as well as Greek local germplasm (Tables S1–S4). The proportion of the harvested area in Greece represented by the studied cultivars was estimated using 2021 data provided by OPEKEPE.^18^

Fruit samples were collected from mature trees cultivated in experimental orchards at the Department of Deciduous Fruit Trees in Naoussa, Greece (40° 37′ 13.40′′ N, 22° 06′ 59.80′′ E; 119 m.a.s.l.). Sampling took place during the 2019 growing season for the apricot cultivars and the 2020 growing season for the peach cultivars. For peach, fruit from four cultivars (‘PI‐A39’, ‘Vlg’, ‘Everts Selection’, and ‘Andros Late’) was collected from nearby orchards, while for apricot, fruit from five local cultivars was collected from other regions: ‘Pr. Tirynthos’ and ‘Diamantopoulou’ (Chalkidiki), ‘Ydroussas’ and ‘Ag. Konstantinou’ (Samos island), and ‘Kaisi Ikarias’ (Ikaria island). Climatic conditions prevailing during 2019 and 2020 have been previously characterized in relation to the split‐pit incidence in melting peach and nectarine cultivars.^10^ Mean temperature between 15 May and 15 September averaged 24.9 °C in 2019 and 24.0 °C in 2020, while cumulative rainfall totaled 146 and 216 mm, respectively. The soil is sandy‐loam, neutral reaction (pH 6.8), ‘low’ carbonate content and 2.5% of organic matter (average measures from soil depth to 45 cm).

Peach trees were grafted on a GF677 rootstock, and apricot trees were grafted on Μyrobolan 29C rootstock. Trees were planted in a 3.5 m × 5 m distance in 3–6 replicates and trained in a vase system. Trees received routine horticultural care with fertilization, pest control, and irrigation.

The studied peach cultivars were characterized according to their type, as MPE (46), NE (25), NMPE (25), and FLAT (4) and according to their flesh color they were yellow‐ or white‐fleshed (77 and 23 cultivars, respectively). The peach cultivars originate from Canada (1), France (15), Greece (5), Italy (19), Spain (8), and USA (47) while 5 cultivars were of unknown origin (Tables S1–S3). The apricot cultivars originate from France (12), Greece (13), Italy (6), and Spain (1) (Table S4).

The fruit characteristics were measured on a 30‐fruit sample per cultivar harvested at the commercial stage based on size, color, and firmness. Fruits were selected for their uniformity in size, color, and lack of defects. RD was expressed as Julian Day (JD).

### Fruit physical traits

Upon harvesting, the fruit fresh weight (FFW) was determined with a digital precision scale. Peel color was measured on the surface (ground peel color) using a Chromo meter (Minolta CR‐200, Ramsey, NJ, USA), equipped with an 8 mm measuring head and a Cilluminant (6774 K). The meter was calibrated using the manufacturer's standard white plate. Color was quantified in the L*, a*, and b* color space. L* refers to the lightness, ranging from black = 0 to white = 100, a* ranges from −a* = greenness to +a* = redness, and b* ranges from −b* = blueness to +b* = yellowness. Readings were also taken in the mesocarp after cutting the fruit into slices.

The amount of extractable juice, indicating free moisture, was quantified by processing 100 g of pulp tissue from three replicates of four fruits using an electrical liquidizer (AE1917 Bomann, Germany), which separated the liquid from the solid fraction. The weight of the recovered juice was expressed as a percentage of the FFW.

For apricot only, the percentage of fruit having a red blush was assessed visually and expressed as %red peel.

### SSC and TA

SSC and TA were measured in the juice obtained for the extractable juice assessment. SSC was quantified with the employment of a refractometer (Atago, PR‐32*α*, Japan) and results expressed as °Brix. TA was measured using an automatic titrator (Titrometic 25; Crison Instruments SA, Barcelona, Spain) by titrating 5 mL of juice diluted in 45 mL of distilled water with 0.1 N NaOH to a pH endpoint of 8.1. Results were expressed as g malic acid L^−1^. RI was calculated as the SSC/TA ratio.

### Polyphenol and carotenoid determinations and antioxidant capacities

Measurements of total phenolics (TPs), total antioxidant capacity (TAC), and total carotenoids (the latter only in apricot) were conducted on a dry weight (DW) basis. Two wedge‐shaped slices from the intact fruit were dissected, and immediately frozen into liquid nitrogen and stored at −20 °C for 24 h. Subsequently, they were dehydrated using a freeze dryer. The dried material was then ground under liquid nitrogen with a mortar and pestle to obtain a fine powder. This powder was stored at −20 °C until further analyses.

Frozen samples (about 0.4 g) were homogenized in 8 mL of 80% (v/v) MeOH–H_2_O using a mortar and pestle. The extract was centrifuged at 10 000 × *g* for 10 min, and the supernatant was recovered.

The TP content was measured using the Folin–Ciocalteu colorimetric method.^19^ The reaction mixture consisted of 0.3 mL of diluted extract, 0.2 mL of distilled water, and 2.5 mL of 10% Folin–Ciocalteu reagent. The tube was vortexed and then allowed to stand at room temperature for 3 min while 2 mL of saturated sodium carbonate solution was added. The solution was incubated for 5 min at 50 °C, and the absorbance was measured at 760 nm against a blank solution. Each measurement was repeated in duplicate. TP content was expressed as mg gallic acid equivalents (GAE) per 100 g DW.

The TAC was evaluated using 1,1‐diphenyl‐2‐picrylhydrazyl (DPPH) (TAC_DPPH_) and ferric reducing antioxidant power (FRAP) (TAC_FRAP_) assays, and they were performed as described by Drogoudi *et al*.^20^

For the DPPH assay, reaction mixtures containing 0 or 20 μL of diluted MeOH extract, 2.3 mL of 106.5 μmol L^−1^ DPPH in MeOH, and 680 μL of water were vortexed and then kept at room temperature in darkness for 4 h.^20^ The absorbance of each reaction mixture was measured at 517 nm.

For the FRAP assay, a sample containing 3 mL of freshly prepared FRAP solution (0.3 mol L^−1^ acetate buffer (pH 3.6) containing 10 mmol L^−1^ 2,4,6‐tripyridyl‐*s*‐triazine and 40 mmol L^−1^ FeCl_3_⋅10H_2_O) and 20 μL of peel or 50 μL of flesh extract was incubated at 37 °C for 4 min, and the absorbance was measured at 593 nm.

A standard curve was obtained on each measurement day, using ascorbic acid standard solution, and accordingly, the results are expressed as milligrams of ascorbic acid (ASC) equivalents per 100 g DW.

Total carotenoids were assayed using the procedure described by Kuti.^21^ Dry weight samples (about 0.2 g) were homogenized in 1/1/2 ethanol–acetone–*n*‐hexane solution using a pestle and mortar. After it was well shaken, the extract was allowed to stand for about 30 min, and the absorbance of the upper layer of hexane was measured at 450 nm using a spectrophotometer. The total carotenoid content was calculated using an extinction coefficient of *β*‐carotene *E* ^1%^ = 2592 and expressed as micrograms of carotene per 100 g DW.

### Statistical analyses

Mean, minimum, and maximum values, and coefficient of variation (CV) were calculated for all evaluated phenotypic traits. In peach, the data were analyzed using a two‐way analysis of variance (ANOVA), considering fruit type (FLAT, MPE, NE, and NMPE) and flesh color (yellow or white) as fixed factors. When the ANOVA *F*‐test was significant (*P* < 0.05), treatment means were compared using Duncan's multiple range test.

To assess the distribution of observations within predefined intervals, percentage frequency distributions were calculated. Each trait range was divided into five classes: ‘low’, ‘low–medium’, ‘medium’, ‘medium–high’ and ‘high’. Frequency distribution analyses were conducted separately for MPE, NE, and NMPE cultivars, while the FLAT type, represented by only four cultivars, was included within the MPE group. Pearson's correlation coefficients were calculated to assess relationships among traits.

Principal component analysis (PCA) was applied to the mean values of the measured traits. Hierarchical clustering of both rows and columns was carried out using Euclidean distance and Ward's method. Statistical analyses were performed using SPSS, version 13.0 (SPSS Inc., Chicago, IL, USA). Additionally, PCA and heatmap visualizations were generated using the ClustVis online platform, with clustering based on Euclidean distance and Ward's method.

## RESULTS AND DISCUSSION

### Fruit quality traits in peach cultivars

The studied peach cultivars include traditional and local cultivars, accessions, and recently released cultivars. Among them, 50 of the 75 table peach cultivars (MPE and NE) and 12 of the 25 NMPE cultivars are widely cultivated in Greece. Collectively, these cultivars represent a moderate proportion of the harvested area for MPE and NE (50.2–60.7%) and the majority for NMPE (93.2%) (Tables S1–S3).^18^ The main MPE cultivars were ‘Royal Glory’, ‘Sweet Scarlet’, and ‘O'Henry’ (10.3%, 6.7%, and 4.6% of the harvested area, respectively); the leading NE cultivars were ‘Big Bang’, ‘Big Top’, and ‘Orion’ (16.8%, 10.3%, and 9.7%, respectively); and the principal NMPE cultivars were ‘Andross’, ‘Catherina’, ‘PI‐A37’, and ‘Everts’ (39.0%, 24.6%, 13.0%, and 12.8%, respectively). Information on the key quality traits of the cultivars studied may support cultivar selection and inform market‐oriented promotion strategies.

**Figure 1:**
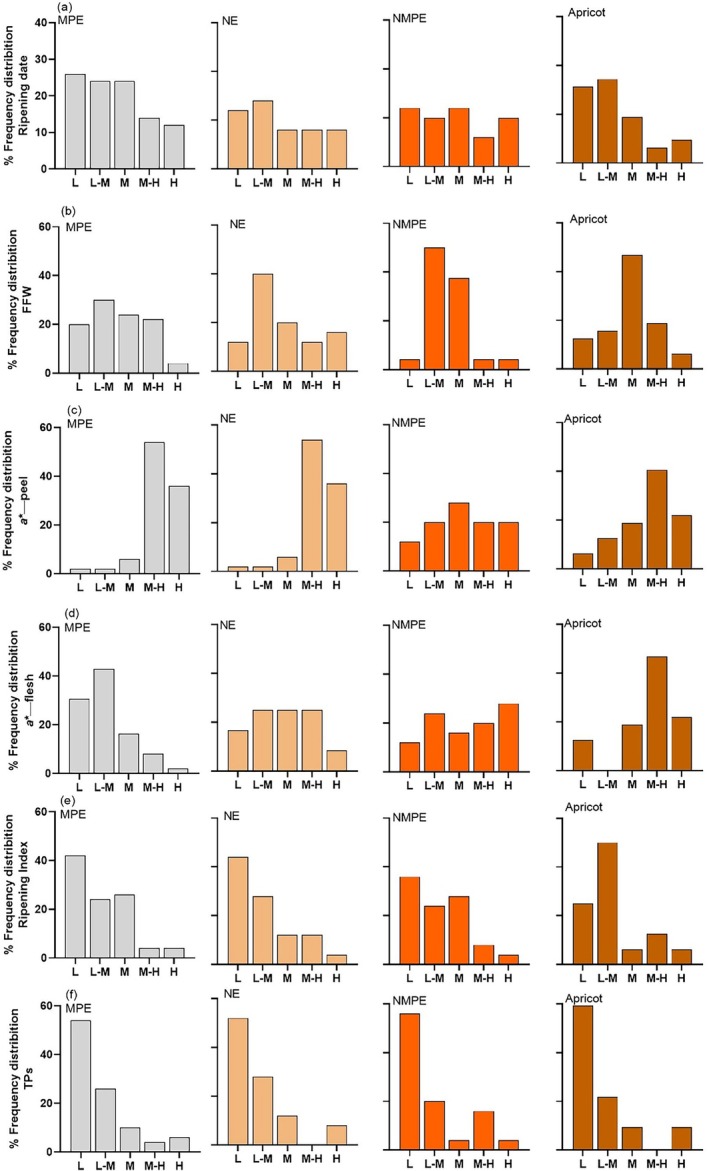
Frequency distributions of (a) RD, (b) FFW, (c) a* peel and (d) a* flesh color parameters, (e) RI, and (f) TP in 50 MPE, 25 NE, 25 NMPE, and 32 apricot cultivars. Values are grouped into five categories based on observed ranges: low (L), low–medium (L–M), medium (M), medium–high (M–H), and high (H).

Table peach cultivars ripened between 29 May and 12 September (148–254 JD) (Table 1; Fig. 1(a)), corresponding to the range of commercially available cultivars in the region and coinciding with a period of favorable climatic conditions in northern Greece. In contrast, NMPE cultivars began ripening approximately 1 month later, starting on 26 June. This pattern is consistent with the cultivars that are currently available,^22^ which enables the canning industry to begin processing later in the season, after apricot fruit is used for canning.

**Table 1:** Summary statistics (mean, range, fold change, and CV) for physical and chemical traits measured in 100 peach and 32 apricot cultivars

	Peach				Apricot			
	Mean	Range	Fold	CV (%)	Mean	Range	Fold	CV (%)
Physical traits								
RD	197	148–254	1.7	14.4	168	142–212	1.5	11.8
FW	203.9	95.6–360.7	3.8	27.5	78.1	33.1–128.2	3.9	26.3
% Red peel	−	−	−	−	18.0	4.0–41.9	10.4	48.9
*L* peel	48.6	28.9–67.6	2.3	23.3	56.3	44.9–70.4	1.6	8.8
a* peel	15.2	−3.8 to 27.5	9.2	53.1	11.2	−4.2 to 20.7	6.4	54.2
b* peel	21.6	7.7–37.5	4.9	38.9	28.2	20.7–32.7	1.6	8.9
*L* flesh	62.9	49.9–74.7	1.5	6.2	55.2	49.1–64.7	1.3	6.9
a* flesh	−0.7	−7.4 to 9	3.2	620.7	10.1	0.2–15.9	68.3	37.1
b* flesh	28.8	12.6–37.7	3	21.8	26.7	23.3–30	1.3	6
% Juice	46.3	31.9–69.6	2.2	14.5	49.3	31.6–61.5	1.9	12.6
Chemical traits								
SSC	12.6	8.9–17.3	1.9	13.5	12.9	8.5–17.5	2.1	17.3
ΤΑ	1.2	0.5–2.3	4.6	32	2.2	1.1–3.3	3.1	25.6
RI	11.6	5–26.4	5.3	38	6.4	3.5–12.2	3.4	32.6
TPs	484.9	213.3–1376.3	6.5	53.4	355.0	149.2–989.6	6.6	64.4
TAC_DPPH_	302.4	86.7–985.3	11.3	61.5	326.8	56.4–1062.8	18.8	75.7
TAC_FRAP_	426.1	59.3–1266.7	21.5	65	253.1	77–782.7	10.2	67.7
TC		−	−	−	92.9	29.9–170.3	5.7	42.2

RD, ripening date (Julian date); FW, fruit fresh weight (g); SSC, soluble solid content (°Brix); TA, titratable acidity (g malic acid per 100 mL juice); RI, ripening index (SSC/TA); TPs, total phenols (mg gallic acid equivalent per 100 g dry weight); TAC_DPPH_ and TAC_FRAP_, total antioxidant capacity using the DPPH and FRAP radicals, respectively (mg ascorbic acid equivalent per 100 g dry weight); TC, total carotenoids (μg carotene per 100 g dry weight).

FFW showed considerable variability, with a 3.8‐fold difference among cultivars (Table 1), with values skewed to ‘low–medium’ and ‘medium’ categories mainly in NMPE cultivars (Table 2; Fig. 1(b)). MPE cultivars exhibited the highest FFW (226.7 g), intermediate values were found in NE, and lower FFW was found in NMPE and FLAT peaches (148.5–177.1 g) (Table 3; Fig. S1). Conversely, no significant differences were observed between yellow‐ and white‐fleshed cultivars. Similar results were reported in our previous study on 58 MPE and NE cultivars studied during 2017–2020.^10^ In the present study thinning was applied at the same intensity in all cultivars, and therefore differences in FFW may be linked to differences in mesocarp development and genetic background.

**Table 2:** Skewness values for MPE (*n* = 50), NE (*n* = 25), NMPE (*n* = 25), and apricot (*n* = 32) cultivars

	MPE	NE	NMPE	Apricot
Physical traits				
RD	0.35	0.34	0.28	0.86
FFW	0.21	0.58	0.99	0.00
Red peel %	−	−	−	0.98
*L* peel	0.47	0.26	0.04	0.48
a* peel	−1.95	−0.42	0.17	−0.64
b* peel	0.46	0.39	−0.22	−0.79
*L* flesh	0.49	−0.47	0.49	0.48
a* flesh	0.91	−0.08	−0.28	−1.03
b* flesh	−0.63	−1.05	0.17	0.09
% Juice	0.42	−0.05	−0.04	−0.65
Chemical traits				
SSC	−0.09	0.33	0.08	−0.23
TA	0.27	0.39	−0.14	0.21
RI	0.75	1.15	0.96	1.10
TP_S_	1.64	1.59	1.06	1.88
TAC_DPPH_	1.56	1.48	0.94	1.76
TAC_FRAP_	1.18	1.25	0.71	1.75
TC	−	−	−	0.20

Moderate to high skewness values (i.e. <−0.5 or >0.5) are highlighted with distinct colors to indicate the direction of skew: negative (left‐skewed) or positive (right‐skewed) distributions.

RD, ripening date (Julian date); FW, fruit fresh weight (g); SSC, soluble solid content (°Brix); TA, titratable acidity (g malic acid per 100 mL juice); RI, ripening index (SSC/TA); TPs, total phenols (mg gallic acid equivalent per 100 g dry weight).

**Table 3:** *P* values from two‐way ANOVA testing the effects of fruit type (MPE, NE, NMPE, and FLAT) and flesh color (yellow and white) on physical and chemical traits of peach fruit

	Type	Color	Type × color
Physical traits			
RD	0.190	0.883	0.590
FFW	**0.001**	0.332	0.848
*L* peel	**0.000**	0.438	0.221
a* peel	**0.000**	0.601	0.264
b* peel	**0.000**	0.133	0.255
*L* flesh	**0.032**	**0.007**	0.581
a* flesh	**0.000**	**0.028**	**0.019**
b* flesh	**0.017**	**0.000**	0.765
% Juice	**0.030**	0.466	0.911
Chemical traits			
SSC	**0.012**	0.664	0.114
ΤΑ	**0.004**	0.073	**0.007**
RI	**0.011**	0.059	0.085
TPs	0.138	0.524	0.136
TAC_DPPH_	0.659	0.469	0.358
TAC_FRAP_	0.105	0.519	0.181

Significant effects (*P* < 0.05) are shown in bold.

RD, ripening date (Julian date); FW, fruit fresh weight (g); SSC, soluble solid content (°Brix); TA, titratable acidity (g malic acid per 100 mL juice); RI, ripening index (SSC/TA); TPs, total phenols (mg gallic acid equivalent per 100 g dry weight).

Color traits showed considerable variability across cultivars, with the highest CVs (39–53%) observed for a* peel and b* peel parameters, reflecting large differences in red and yellow pigmentation (Table 1). In MPE and NE cultivars, the distribution of a* peel values was skewed toward higher redness, reflecting recent market preference for highly colored fruit (Table 2; Fig. 1(c)). In NMPE cultivars, peel coloration was characterized by higher lightness (greater L*), reduced redness (lower a*), and increased yellowness (higher b*). For a* flesh, a higher proportion of cultivars were classified in the ‘low’ and ‘low–medium’ categories only in MPE (more being yellow‐fleshed), coinciding with having commercial importance and market acceptance.^22^ In NMPE cultivars, the flesh exhibited significantly greater redness (higher a*), compared to MPE and NE cultivars (Table 3; Fig. S1). This contrast highlights a pronounced differentiation between peel and flesh coloration in NMPE cultivars, aligning with previously reported observations.^20^, ^23^

In the present study, juice percentage varied 2.2‐fold among peach cultivars, ranging from 31.9% to 69.6%, with an approximately normal distribution (Tables 1 and 2), consistent with previous reports on peach juice content.^24^ Cultivars with relatively high juice content were ‘Zee Lady’ from MPE, ‘Big Top’ and ‘Honey Royal’ from NE, and ‘Mirel’ from NMPE group of cultivars (Figs 3, 4, 5). As expected, the NMPE cultivars exhibited the lowest juice yield (43.7%), whereas MPE, NE, and FLAT types showed higher extractability (44.6–48.7%) (Table 3; Fig. S1). These differences likely reflect structural differences in mesocarp tissue, as NMPE fruit maintain firmer cell adhesion and reduced cell wall degradation during ripening, limiting juice release. No significant differences in juice yield was observed between yellow‐ and white‐fleshed peaches. Given the strong positive relationship between juiciness and consumer acceptance reported previously,^25^ juice content remains a key quality attribute, with additional relevance for juice processing. The common perception that older cultivars are ‘juicier’ may reflect historical breeding trade‐offs favoring increased firmness, alongside improvements in color and fruit weight. Reintroducing the juiciness traits into modern breeding programs could improve consumer satisfaction.

SSC ranged from 8.5 to 22.2 °Brix among the cultivars studied (Table 1). Being a general quality criterion, SSC should be higher than 10 °Brix and 11 °Brix for early‐ and mid‐/late‐ripening cultivars, respectively.^26^ Moreover SSC greater than 12 °Brix was recently found as a prerequisite to perceive aroma in peach cultivars.^5^ TA ranged from 0.3 to 2.3 g (100 mL)^−1^ and RI spanned from 5.0 to 42.7 (Table 1). FLAT (16.1) followed by NMPE (13.3) showed the highest RI, while lowest values were found in MPE and NE cultivars (10.8) and white‐fleshed peaches exhibited higher RI than yellow‐fleshed types (12.7 *versus* 11.3, *P* = 0.016) (Table 3; Fig. S1), a finding similarly reported in our previous study where 45 peach and nectarine cultivars were analysed.^19^ This variation in SSC and TA across texture and color types underscores the need to consider these traits when selecting cultivars for fresh consumption.

Overall, mean TP content was higher in peach than in apricot (484.9 *versus* 355.0 mg (100 g)^−1^ DW). However, as measurements in each species were conducted in different years, climatic variation may limit interspecific comparisons.^17^ In peach, an important variation in TP contents was found (6.5‐fold) with values ranging from 213.3 to 1376.3 mg GAE (100 g)^−1^ DW and the frequency distribution was highly skewed in all peach types towards lowest values (skewness 1.06–1.64); 52–56% was in the ‘low’ category (Tables 1 and 2; Fig. 1(f)). Although the effect of fruit type on antioxidant content was not statistically significant, a trend was observed in TP concentrations, with higher mean values in MPE (524.2 mg (100 g)^−1^ DW), followed by NE (481.4 mg (100 g)^−1^ DW), and lower levels in NMPE (409.6 mg (100 g)^−1^ DW) (*P* = 0.090) (Table 3; Fig. S1). This pattern aligns with previous studies that reported higher TP levels in MPE compared to NE cultivars.^27^, ^28^ Similarly, lower TP content in NMPE relative to MPE cultivars has been reported.^7^ No significant differences in antioxidant content were observed among flesh color groups; despite earlier findings suggesting higher antioxidant concentrations in white‐fleshed peaches.^7^, ^27^

Among MPE cultivars, the highest TP concentrations were observed in ‘Summer Lady’ and ‘Corridon’ (1311.3–1376.3 mg (100 g)^−1^ DW). In NE cultivars, ‘Max’ and ‘Late Fair’ exhibited the greatest values (1180.7–1303.0 mg (100 g)^−1^ DW), whereas among NMPE cultivars, ‘Fercluse’, ‘Farlate’, and ‘Quadelupe’ showed the highest levels (730.0–875.0 mg (100 g)^−1^ DW) (Figs 3, 4, 5). Notably, ‘Fercluse’ and ‘Ferlate’ were previously separated as the canned product having the greatest firmness retention after 24 months of storage, an important traits for the canning industry.^29^ A comparable range of TPs (117–901 mg GAE (100 g)^−1^ DW) was reported in our previous study, where cultivars exhibiting the highest levels included ‘Sun Cloud’, ‘Gladys’, ‘Sun Crest’, ‘Opsimo Naoussas’, ‘Fayette’, and ‘Rubidoux’ that were not included in the present study.^19^

In the present study TAC_DPPH_ ranged 11.3‐fold (from 86.7 to 985.3 mg ASC equivalent per 100 g DW) and TAC_FRAP_ ranged 21.3‐fold (from 59.3 to 1266.7 mg ASC equivalent per 100 g DW). Most cultivars were in the ‘low’ and ‘low–medium’ categories of fruit antioxidant contents, similar to the TP contents (Tables 1 and 2).

### Fruit quality traits in apricot cultivars

The cultivars examined in this study account for 42.5% of the total harvested apricot area in Greece. Among them, the traditional canning cultivar ‘Bebecou’ predominates, followed by ‘Pr. Tirynthos’, ‘Farbaly’, and ‘Ladycot’, representing 25.1%, 7.9%, 6.9%, and 4.2% of the harvested area, respectively (Table S4). The apricot fruit ripening period ranged from 23 May to 8 August (142–212 JD), beginning approximately 1 week earlier and ending 1 month earlier than that of the peach cultivars (Table 1). The distribution of RD was skewed toward early ripening (skewness = 0.86), with most apricot cultivars ripening by 19 June (170 JD) and thus classified as precocious (Table 2; Fig. 1(a)). Earliest ripening was found in cultivars ‘Mogador’, ‘Spring Blush’, ‘Tsunami’, and ‘Wondercot’ and latest ripening was in ‘Fardao’, ‘Farhial’, and ‘Congat’ (Fig. 6).

Important variation was found in the apricot FFW (3.9‐fold, CV = 26.3%) (Table 1); the lowest values were recorded in the Greek traditional cultivar ‘Diamantopoulou’ and the early harvesting ‘Spring Blush’, whereas ‘Farbaly’ and ‘Bora’ were separated for highest FFW. Substantial variability was also observed for peel and flesh color traits, as reflected by high coefficients of variation for a* peel (54.2%) and a* flesh (37.1%). As observed in peach, the frequency distributions were skewed toward redder peel and flesh coloration (skewness = −0.79 and −1.03, respectively) (Table 2; Fig. 1(c),(d)). The highest a* peel values were found in ‘Big Red’ and ‘Ladycot’, while the highest a* flesh values were found in ‘Bora’, ‘Ladycot’, and ‘Spring Blush’. In addition, the red peel coverage showed marked variation (CV = 48.9%), with ‘Tsunami’ and ‘Ag. Constantinou’ displaying the highest red peel coverage (41.9% and 38.4%, respectively).

Percentage juice varied nearly twofold among the apricot cultivars, with most clustering in the ‘medium–high’ category (40.7%), and a moderately left‐skewed distribution (skewness = −0.65) (Table 2). This pattern indicates that relatively high juice content is a common trait within the evaluated germplasm. The highest juice yields were observed in ‘Mogador’, ‘Bergeval’, and ‘Bebecou’ (58.9–61.5%), whereas ‘Farhial’, ‘Farely’, and ‘Fardao’, the late ripening cultivars, exhibited the lowest values (31.6–40.7%).

The values recorded for SSC in the present study were between 8.5 and 17.5 °Brix, with cultivars ‘Congat’, ‘Fardao’, and ‘Farhial’ exhibiting the highest values. The above values were in good accordance with those reported in the literature for cultivars being similarly studied, showing also relatively high SSC in ‘Farhial’.^30^ Fruit TA content was between 1.1 and 3.3 mg (100 g)^−1^; both traits followed normal distributions (Tables 1 and 2). RI spanned from 3.5 to 12.2, with half of the cultivars classified in the ‘low–medium’ category (50%; skewness = 1.095) (Fig. 1(e)). The highest RI values were observed in the late ripening and of French origin cultivars ‘Farely’, ‘Congat’, ‘Fardao’, and ‘Farhial’, whereas the lowest values occurred in the Italian genotypes ‘AB4’, ‘Bora’, and the early ripening and Greek origin ‘Pr. Tirynthos’, largely associated with relatively elevated TA levels.

Fruit antioxidant traits exhibited the highest variability among all evaluated parameters. TPs showed a 6.6‐fold variation, ranging from 142 to 990 mg GAE (100 g)^−1^ DW. TAC_DPPH_ and TAC_FRAP_ assays varied by 10.2‐ and 18.8‐fold, respectively (56–1063 and 77–783 mg ASC equivalents per 100 g DW) (Table 1). The frequency distributions of antioxidant traits were strongly skewed toward lower values (skewness = 1.754–1.884), with 46.9–59.4% of cultivars classified in the ‘low’ category (Table 2; Fig. 1(f)). The highest TP contents were recorded in ‘Farhial’, ‘Congat’, and ‘AB4’ (926–985 mg (100 g)^−1^ DW), whereas the lowest values were observed in ‘AB5’ and ‘Ag. Constantinou’ (*ca* 149 mg (100 g)^−1^ DW) (Fig. 6). These ranges are consistent with values previously reported for apricot germplasm,^14^, ^31^, ^32^ yet a pronounced variation in the studied cultivars was recorded in the present study. The apricot ‘Farhial’ was similarly separated for high TPs in the study by Kafkaletou *et al*.^30^ where eight apricot cultivars were analyzed.

Total carotenoid content exhibited an almost sixfold variation among apricot cultivars and values followed a normal distribution (Tables 1 and 2). The highest concentrations were recorded in ‘Ag. Constantinou’, ‘Diamantopoulou’, and ‘Ydroussas’ (170.3, 153.5, and 152.5 mg *β*‐carotene per 100 g DW, respectively), whereas the lowest levels were observed in ‘Farhial’, ‘AB5’, and ‘Fardao’ (36.6, 33.3, and 29.9 mg *β*‐carotene per 100 g DW) (Fig. 6). The mean total carotenoid values observed were comparable to those reported in previous studies.^13^, ^30^, ^33^ In particular, the cultivars ‘Diamantopoulou’ and ‘Farhial’ were similarly previously distinguished with the former exhibiting relatively high carotenoid content and the latter among the lowest.^30^

### Correlations among variables

Most significant correlations among the assessed physical and chemical fruit quality are summarized in Table 4, while a full set of correlation analyses, encompassing all measured traits, is presented in Figs [Link], [Link].

**Table 4:** Heat map showing Pearson correlation (*r*) coefficients between separated pairs related to fruit physical and chemical traits of MPE, NE, NMPE, and apricot cultivars^a^

		MPE	NE	NMPE	Apricot
RD	RD *vs*. FFW	0.662**	0.518**	ns	ns
	RD *vs. L* peel	0.458**	0.860**	ns	ns
	RD vs. a* peel	ns	−0.757**	ns	ns
	RD vs. b* peel	0.549**	0.805**	−0.582**	ns
	RD *vs. L* flesh	0.288*	0.472*	0.509**	ns
	RD vs. a* flesh	0.442**	0.534**	−0.522**	ns
	RD vs. b* flesh	ns	ns	0.511**	ns
	RD *vs*. % juice	ns	ns	ns	−0.524**
	RD *vs*. SSC	0.760**	0.766**	ns	0.597**
	RD *vs*. TA	−0.500**	ns	−0.680**	ns
	RD *vs*. RI	0.617**	ns	0.547**	0.553**
	RD *vs*. TPs	0.355*	0.619**	ns	0.549**
	RD *vs*. TAC_DPPH_	0.357*	0.606**	ns	0.548**
	RD *vs*. TAC_FRAP_	0.329*	0.633**	ns	0.505**
Color	L vs. b* peel	0.877**	0.956**	0.783**	0.613**
	L vs. a* peel	−0.671**	−0.798**	ns	−0.451**
	a* vs. b* peel	−0.540**	−0.755**	ns	ns
	L vs. b* flesh	−0.307*	ns	0.713**	ns
	L vs. a* flesh	ns	ns	−0.590**	−0.444*
	a* vs. b* flesh	ns	0.562**	−0.552**	0.357*
Antioxidants	TPs *vs*. TAC_DPPH_	0.919**	0.981**	0.973**	0.978**
	TPs *vs*. TAC_FRAP_	0.957**	0.989**	0.983**	0.971**
	TAC_DPPH_ *vs*. TAC_FRAP_	0.944**	0.983**	0.975**	0.969**
	TC *vs*. TPs	**‐**	**‐**	**‐**	ns
	TC *vs*. TAC_DPPH_	**‐**	**‐**	**‐**	ns
	TC *vs*. TAC_FRAP_	**‐**	**‐**	**‐**	ns

^a^ Correlation significant differences: *P < 0.05, **P < 0.01; ns, not significant. Absolute correlation coefficients |*r*| > 0.6 are shown in bold.

RD, ripening date (Julian date); FW, fruit fresh weight (g); SSC, soluble solid content (°Brix); TA, titratable acidity (g malic acid per 100 mL juice); RI, ripening index (SSC/TA); TPs, total phenols (mg gallic acid equivalent per 100 g dry weight).

Strong positive association was found between RD and FFW (*r* = 0.518–0.662), and between RD and SSC (*r* = 0.760–0.766) in table peach cultivars (MPE and NE), as a result of extended maturation period on the tree. Nevertheless, this was not the case for NMPE cultivars, having 1‐month shorter harvesting window. Similar observations were made in previous studies, without a fruit type classification in peach.^5^, ^10^, ^24^, ^27^, ^34^ Apricot cultivars begin ripening as early as May – earlier than peaches – and moderate correlation was found between RI and SSC or RI (*r* = 0.593–0.597). Yet there was no significant correlation between RD and FFW, a finding consistent with the results reported by Badenes *et al*.^35^ in their analysis of 55 apricot cultivars, but contrasts with the outcomes of our previous study conducted on 29 cultivars.^14^ Improving SSC especially in early‐ripening cultivars of table peach and apricot cultivars remains a relevant breeding objective given the established association between RI and consumer acceptance.^36^

In apricot, late‐harvested cultivars were associated with reduced juiciness (*r* = −0.524), in agreement with the findings of Leccese *et al*.^36^, ^37^ As most apricot cultivars ripen early and few are late harvested, these results emphasize the need to develop late‐ripening apricot cultivars that maintain high fruit quality.

In the present study it was found that RD may play a moderately influential role in determining antioxidant accumulation in NE and apricot genotypes (*r* = 0.505–0.633), whereas its contribution appears more limited in MPE and NMPE groups (*r* = 0.329–0.357). However, stronger associations between RD and antioxidant contents were previously reported for peach,^24^, ^33^ but not in Forcada *et al*.,^11^ and apricot cultivars.^33^

Among the color parameters measured in peel tissue, the strongest correlations were observed between brightness (L*) and yellowness (b*). This relationship was consistently high across all peach types (*r* = 0.783–0.956) and was also present in apricot cultivars (*r* = 0.613). In addition, strong negative correlations were detected between peel brightness and redness (L* peel versus a* peel), as well as between redness and yellowness (a* peel versus b* peel), but only in MPE and NE cultivars (*r* = −0.671 to −0.798). These relationships likely reflect the more intense red and blush coloration characteristic of these peach types compared with NMPE peaches and apricots. Overall, these results suggest that, for table peach cultivars, measurements of L* and a* are sufficient to capture variability among cultivars, whereas the b* parameter appears to contribute less additional information.

In contrast, relationships among flesh color traits were generally weak. An exception occurred in NMPE fruit, where lighter flesh was associated with increased yellowness (*L* flesh versus b* flesh, *r* = 0.713), while higher peel redness was linked to lower brightness (a* flesh *versus L* flesh, *r* = −0.590) and lower yellowness (a* flesh versus b* flesh, *r* = −0.552). Overall, these results indicate that color trait relationships in the peel are strongly influenced by blush development, whereas flesh color associations are limited and appear to be specific to NMPE. These findings align with the recent study by Chen and Read,^24^ which reported strong associations between brightness and yellowness but little or no relationship among other color parameters. Notably, the present study extends the previous study by separately analyzing peel and flesh color and by including multiple peach types as well as apricot cultivars.

Strong positive correlations were observed between TPs and both TAC_DPPH_ and TAC_FRAP_, as well as between TAC_DPPH_ and TAC_FRAP_ with correlation coefficients exceeding 0.9 in all peach types and in apricot. These results support a close link between phenolic content and antioxidant capacity in peach^11^, ^27^, ^38^ and apricot^14^ fruit, suggesting that measuring all three parameters simultaneously may be redundant. In contrast, no significant correlations were found between total carotenoid content and antioxidant‐related parameters in apricot. Antioxidant capacity values can be strongly influenced by the extraction method used.^39^ The weak relationship between carotenoids and TAC is likely due to the limited ability of methanolic extraction to recover lipophilic compounds such as carotenoids, while efficiently extracting hydrophilic antioxidants, particularly phenolics. Consequently, TAC measurements mainly reflect the contribution of hydrophilic compounds. Consistent with this interpretation, weak^14^ or no^40^ correlations between phenolic content, antioxidant capacity, and total carotenoid content have been reported previously. Overall, these results demonstrate that phenolic compounds are the primary contributors to measured antioxidant capacity in peach and apricot fruit, whereas carotenoids play a limited role under the extraction and assay conditions used.

Total carotenoid content was not significantly associated with color parameters in the present study, in agreement with previous reports.^17^, ^41^ However, other studies have suggested different outcomes; for example, Ruiz *et al*.^14^ reported that hue angle may be a useful indicator of carotenoid concentration in apricot fruit, indicating that the relationship between color traits and carotenoid content may depend on fruit type or analytical approach.

### Principal component analysis and grouping of cultivars

PCA was conducted using the mean values of the measured traits in order to assess the relative contribution of each parameter to total variation and to elucidate relationships among cultivars and fruit traits within different peach groups and apricot cultivars.

**Figure 2:**
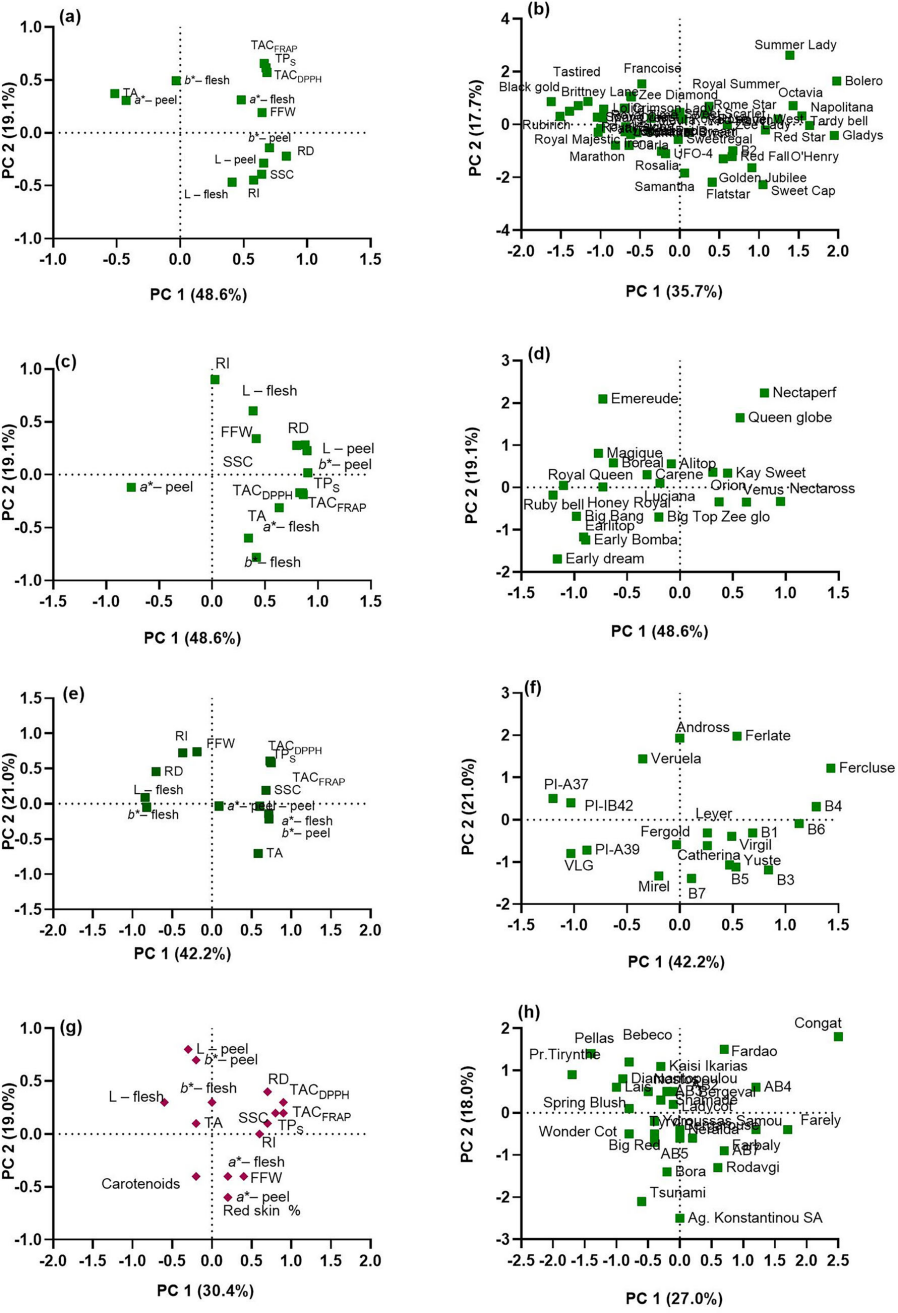
(a, c, e, g) Factor loadings and (b, d, f, h) two‐dimensional space of the first principal component (factor 1) and second principal component (factor 2) of the PCA for (a, b) 50 MPE and FLAT, (c, d) 25 NE, (e, f) 25 NMPE, and (g, h) 32 apricot cultivars, on the basis of fruit physical and chemical characters, determined by PCA. Variable annotations are presented in Table 1.

**Figure 3:**
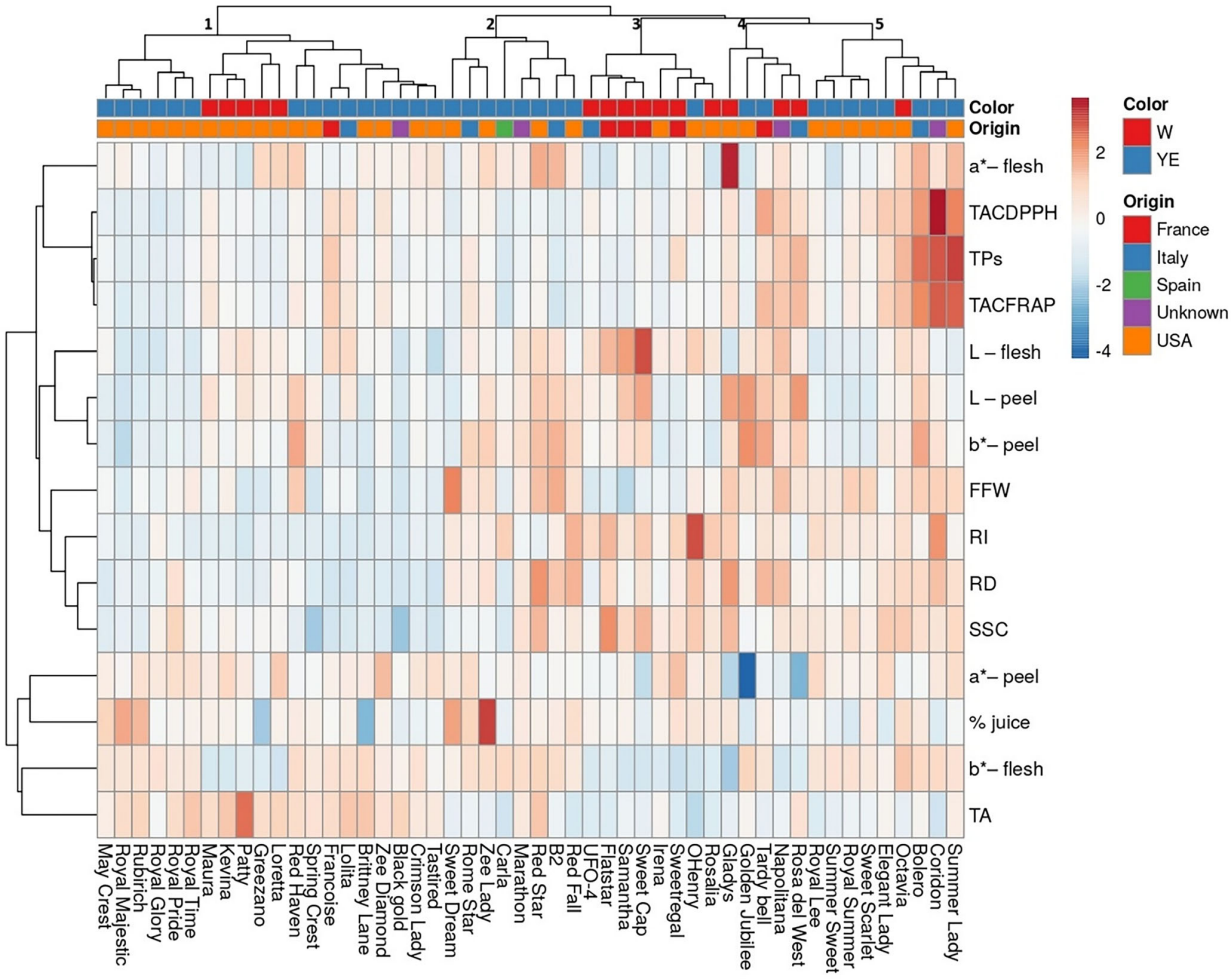
Heatmap showing the clustering of fruit phenotyping traits in 50 MPE cultivars. The columns correspond to the cultivars, and the rows correspond to the fruit phenotypic traits studied. Both rows and columns were clustered using Euclidean distance and the Ward method.

In MPE, the PCA carried out produced four components, with highest contribution by PC1 and PC2 accounting for 35.7% and 17.7% of variance, respectively. The most important variable integrated in PC1 was RD, while a* peel and TA had negative correlations, while PC2 was positively correlated with fruit antioxidant contents and b* flesh (Fig. 2(a)). Cultivars ‘Summer Lady’, ‘Coridon’, and ‘Bolero’ were separated with high antioxidant contents (Fig. 2(b)). Cluster analysis divided the cultivars into four groups; identifying one group (no. 5) with desirable traits such as high antioxidant contents, low TA, high RI, and late RD (Fig. 3).

**Figure 4:**
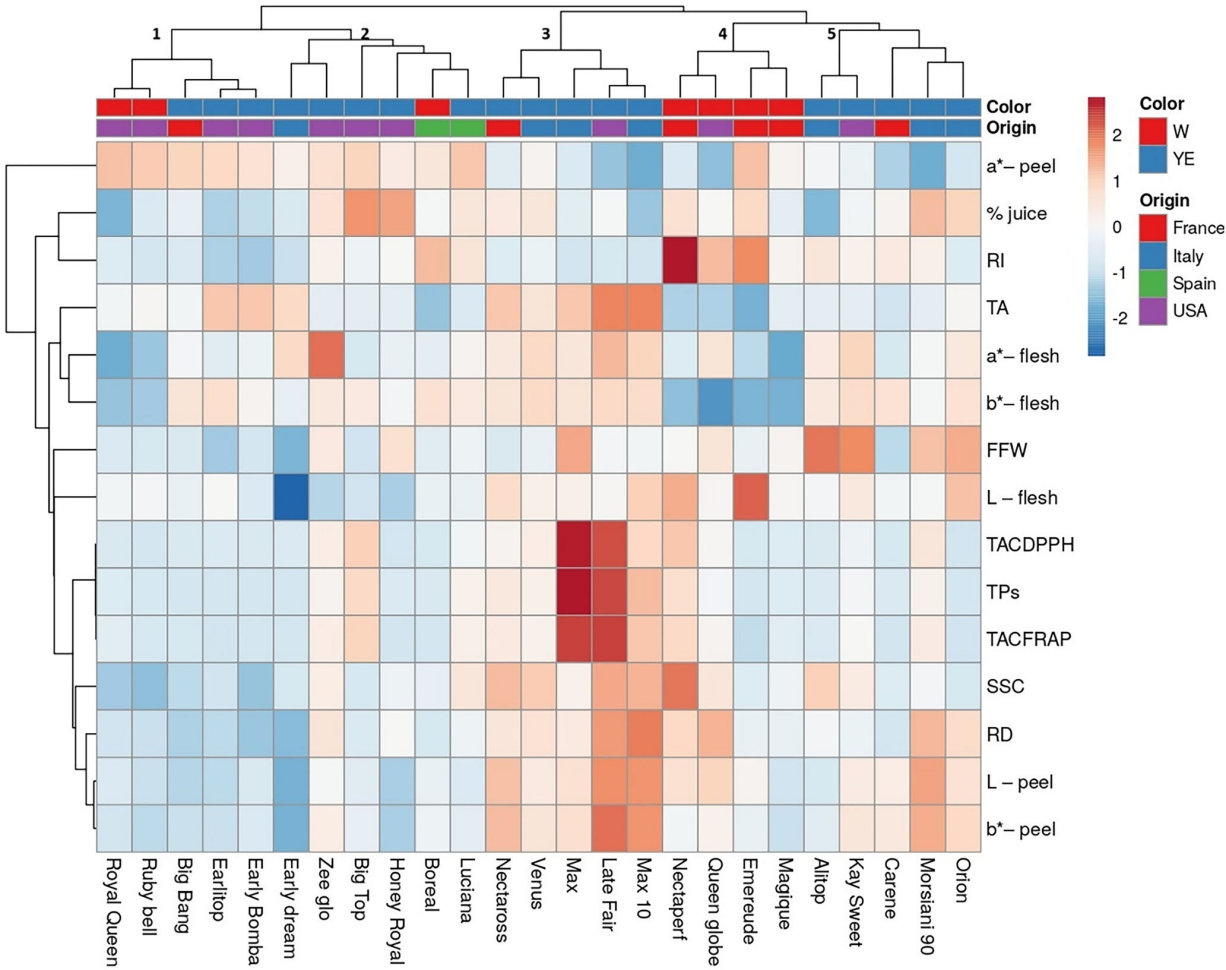
Heatmap showing the clustering of fruit phenotyping traits in 25 NE cultivars. The columns correspond to the cultivars, and the rows correspond to the fruit phenotypic traits studied. Both rows and columns were clustered using Euclidean distance and the Ward method.

In NE, the PCA produced four components: PC1 and PC2 accounted for 48.6% and 19.1% of variance, respectively. The most important variables integrated in PC1 were RD, *L* and b* peel, a* flesh, SSC, and antioxidant contents. PC2 was positively correlated with RI and *L* flesh and negatively with TA (Fig. 2(c)). Cultivars ‘Late Fair’, ‘Max10’, and ‘Max’ were separated with high values in PC1, while cultivars ‘Nectaperf’, ‘Emeraude’, and ‘Queen Globe’ were separated with high values in PC2 (Fig. 2(d)). Cluster analysis grouped the cultivars into five distinct clusters. Cultivars with high antioxidant contents, RD, SSC, and reduced a* peel were separated in cluster no. 3 (Fig. 4).

**Figure 5:**
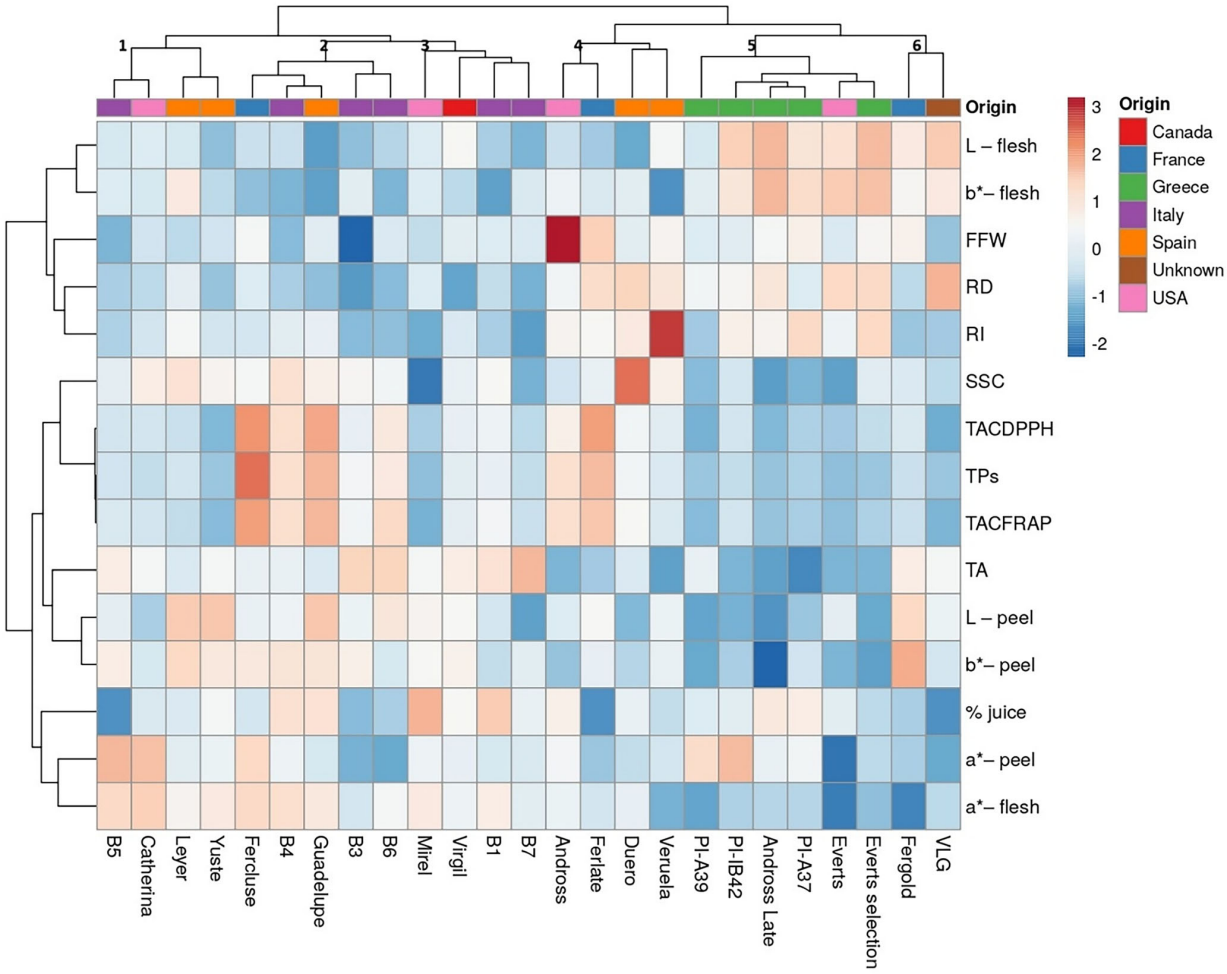
Heatmap showing the clustering of fruit phenotyping traits in 25 NMPE cultivars. The columns correspond to the cultivars, and the rows correspond to the fruit phenotypic traits studied. Both rows and columns were clustered using Euclidean distance and the Ward method.

In NMPE, PCA produced five components: PC1 and PC2 accounted for 42.2% and 21.0% of variance, respectively. The most important variables integrated in PC1 were *L* and *b* peel, *a* flesh, SSC, TA, and antioxidant contents, while negative correlations with *L* flesh and *b* flesh. PC2 was positively correlated with RD, FFW, and RI and negatively with TA (Fig. 2(e)). Cultivars ‘Fercluse’, ‘Quandeloupe’, ‘B4’, and ‘B3’ were separated with high values in PC1 (orange‐color flesh, yellow peel, high SSC and antioxidant contents), whereas cultivars ‘Andross’, ‘Everts’, and ‘Everts selections’ for low values in PC1 (Fig. 2(f)). Cluster analysis divided the traits into three groups, with antioxidant contents showing closer associations with SSC (Fig. 5). Cluster analysis grouped the cultivars into six distinct clusters. Cultivars originating from Greece were separated in cluster no. 5, with high *L* flesh and *b* flesh and lower SSC, antioxidant contents, and *a* flesh values.

**Figure 6:**
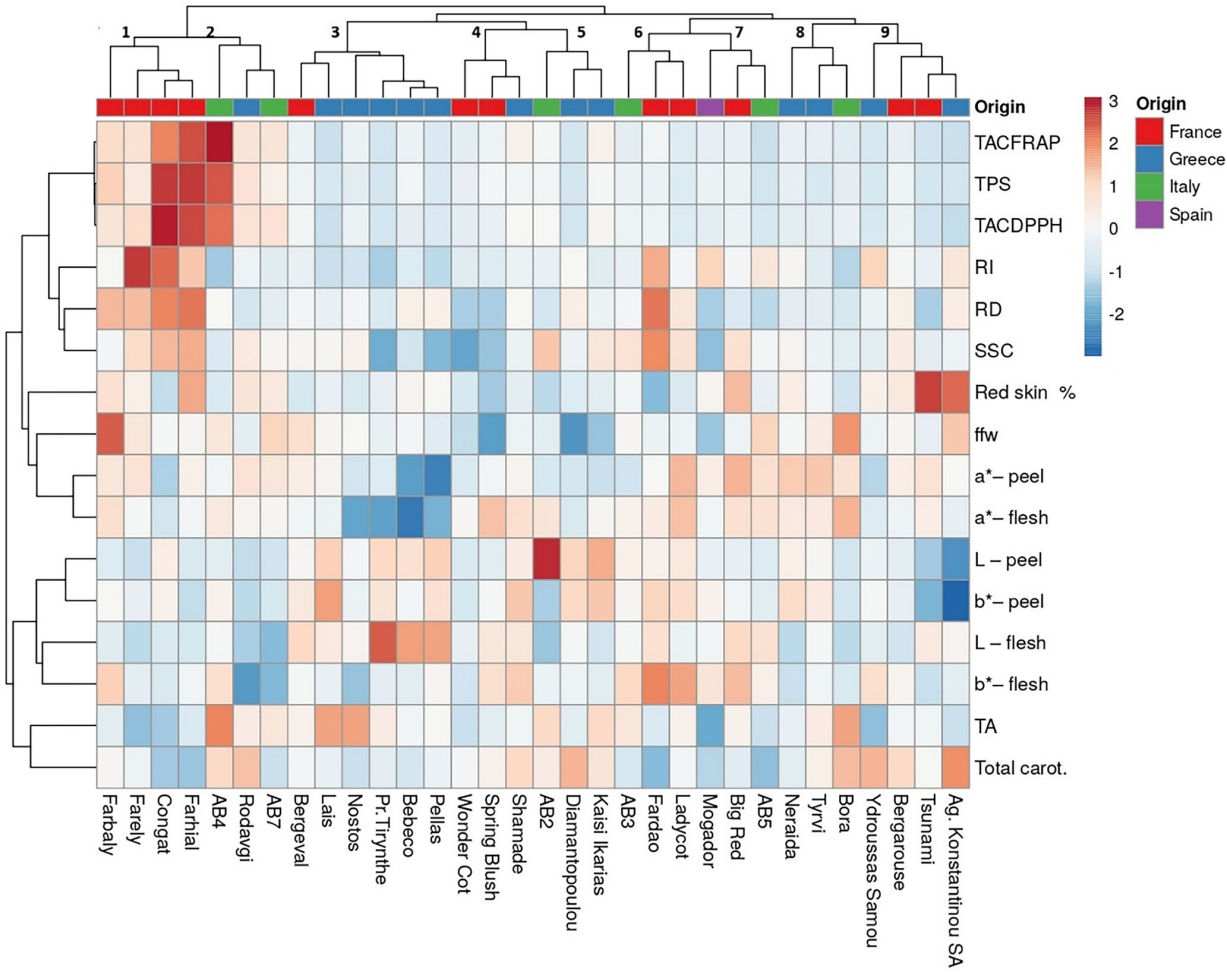
Heatmap showing the clustering of fruit phenotyping traits in 32 apricot cultivars. The columns correspond to the cultivars, and the rows correspond to the fruit phenotypic traits studied. Both rows and columns were clustered using Euclidean distance and the Ward method.

In apricot, the PCA yielded four components: PC1 and PC2 accounted for 27.0% and 18.0% of the total variance, respectively. PC1 was primarily associated with RD, SSC, and antioxidant contents and negatively with *L* flesh. PC2 was positively correlated with L* peel and b* peel and negatively with a* peel and red peel (Fig. 2(g)). Cultivars ‘Farhial’, ‘Congat’, and ‘Farely’ were clearly separated along PC1, reflecting their high values in antioxidant contents, SSC, and late ripeness (Fig. 2(h)). Cluster analysis divided the apricot traits into four groups, with antioxidant contents showing closer associations with RI, RD, and SSC (Fig. 6). Cluster analysis categorized the cultivars into nine distinct groups. Notably, cultivars of French origin formed cluster 1, which was characterized by higher antioxidant contents, later RD, and RI. In contrast, traditional Greek apricot cultivars such as ‘Bebecou’ and ‘Diamantopoulou’ were associated with lower antioxidant levels and reduced peel coloration. These findings are consistent with those reported by Kafkaletou *et al*.,^30^ where Greek cultivars generally exhibited intermediate to low values across most assessed traits and, additionally, French cultivars demonstrated, on average, a sixfold higher sorbitol content compared to those of Greek origin.

## CONCLUSIONS

Substantial phenotypic diversity was observed for fruit quality and antioxidant traits. TP content showed the greatest variation, differing by up to approximately sevenfold among peach and apricot cultivars, while high‐antioxidant genotypes were relatively rare in the evaluated germplasm, highlighting their importance for breeding. Among commercially important cultivars, ‘Octavia’, ‘Roda del West’, and ‘Summer Lady’ (MPE); ‘Big Top’ and ‘Max’ (NE); ‘Andross’, ‘Fercluse’, ‘Ferlate’, and ‘Guadelupe’ (NMPE); and the apricot cultivars ‘Farhial’ and ‘Farbaly’ exhibited relatively high antioxidant levels, having a potential added value for marketing to health‐conscious consumers. Notably, ‘Andross’ (NMPE) and ‘Big Top’ (NE), which account for approximately 39.0% and 10.3% of the cultivated area, respectively, may be considered as having a substantial contribution to the nutritional value of commercially produced fruit in Greece.

A moderate correlation between RD and fruit antioxidant content was observed only in NE and apricot genotypes, whereas its contribution appeared more limited in the MPE and NMPE groups. This pattern suggests that the relationship between these traits may depend on the genetic background and fruit type, indicating that RD should not be regarded as universal predictor of antioxidant accumulation.

Juiciness and RI are two key determinants of consumer acceptance. Juiciness varied nearly twofold in both peach and apricot, while RI differed among peach types, with the highest values observed in FLAT and NMPE cultivars and in white‐fleshed compared with yellow‐fleshed peaches. These results highlight the substantial diversity in texture‐ and ripening‐related traits that can be exploited to improve fruit quality in breeding programs.

Multivariate analyses further demonstrated that RD, RI, peel color parameters, and antioxidant traits were the main drivers of cultivar differentiation, resulting in clear grouping patterns. Cluster analysis in apricot separated French‐origin cultivars forming a group characterized by higher antioxidant contents, late RD, and high RI, whereas cultivars of Greek origin clustered separately and were generally associated with lower antioxidant levels and reduced peel coloration. Overall, the observed diversity provides valuable opportunities for selecting cultivars with desirable sensory attributes and high nutritional value, thereby supporting the development of targeted fruit ideotypes and providing guidance for nutritionally aware consumers.

## AUTHOR CONTRIBUTIONS

PD: Experiment design, data curation, writing, and funding acquisition. GP: Experiment design, laboratory work, and review. ED: laboratory work, writing; KZ: Field and laboratory work. DG: Experiment design, writing and review. All authors have read and agreed to the published version of the manuscript.

## FUNDING INFORMATION

The project was funded by (a) the General Secretariat for Research and Innovation of the Ministry of Development and Investments under the PRIMA Programme. PRIMA is an Art.185 initiative supported and co‐funded under Horizon 2020, the European Union's Programme for Research and Innovation (project code: Prima 2018–03) and (b) the General Secretariat for Research and Innovation of the Ministry of Development and Investments, program Competitiveness, Entrepreneuship and Innovation, under the call RESEARCH–CREATE–INNOVATE (project code: T1EDK‐05438).

## CONFLICT OF INTEREST

The authors declare that the research was conducted in the absence of any commercial or financial relationships that could be construed as a potential conflict of interest.

## Supporting information


**Figure S1.** Mean values (±SE) for the fruit type effects on various physical and chemical traits in 100 peach cultivars. Means with different letters indicate difference among separate treatment means.

**Figure S2.** Heat map of Pearson correlation (r) coefficient matrix between fruit phenotyping traits, in 50 melting peach cultivars. Correlations significant differences: *: *P* < 0.05, **: *P* < 0.01, ***: *P* < 0.001.

**Figure S3.** Heat map of Pearson correlation (r) coefficient matrix between fruit phenotyping traits, in 25 nectarine cultivars. Correlations significant differences: *: *P* < 0.05, **: *P* < 0.01, ***: *P* < 0.001.

**Figure S4.** Heat map of Pearson correlation (r) coefficient matrix between fruit phenotyping traits, in 25 nonmelting peach cultivars. Correlations significant differences: *: *P* < 0.05, **: *P* < 0.01, ***: *P* < 0.001.

**Figure S5.** Heat map of Pearson correlation (r) coefficient matrix between fruit phenotyping traits, in 32 apricot cultivars. Correlations significant differences: *: *P* < 0.05, **: *P* < 0.01, ***: *P* < 0.001.

**Table S1.** Fruit shape, flesh color, origin, and percentage of production area in Greece (2021) for the 50 melting‐flesh peach cultivars studied.
**Table S2.** Flesh color, origin and % production areain Greece (2021) for the 25 nectarine cultivars studied.
**Table S3.** Origin and % production area in Greece (2021) for the 25 nonmelting peach cultivars studied.
**Table S4.** Origin and % production area in Greece (2021) for the 32 apricot cultivars studied.

## Data Availability

The data that support the findings of this study are available from the corresponding author upon reasonable request.
