# Supplementary figures and images for: Large‐scale phenotyping of physical and antioxidant traits in peach and apricot cultivars
Source: J Sci Food Agric. 2026 Apr 16;106(10):6050–63. doi: 10.1002/jsfa.70661 (PMC13258209; doi:10.1002/jsfa.70661)

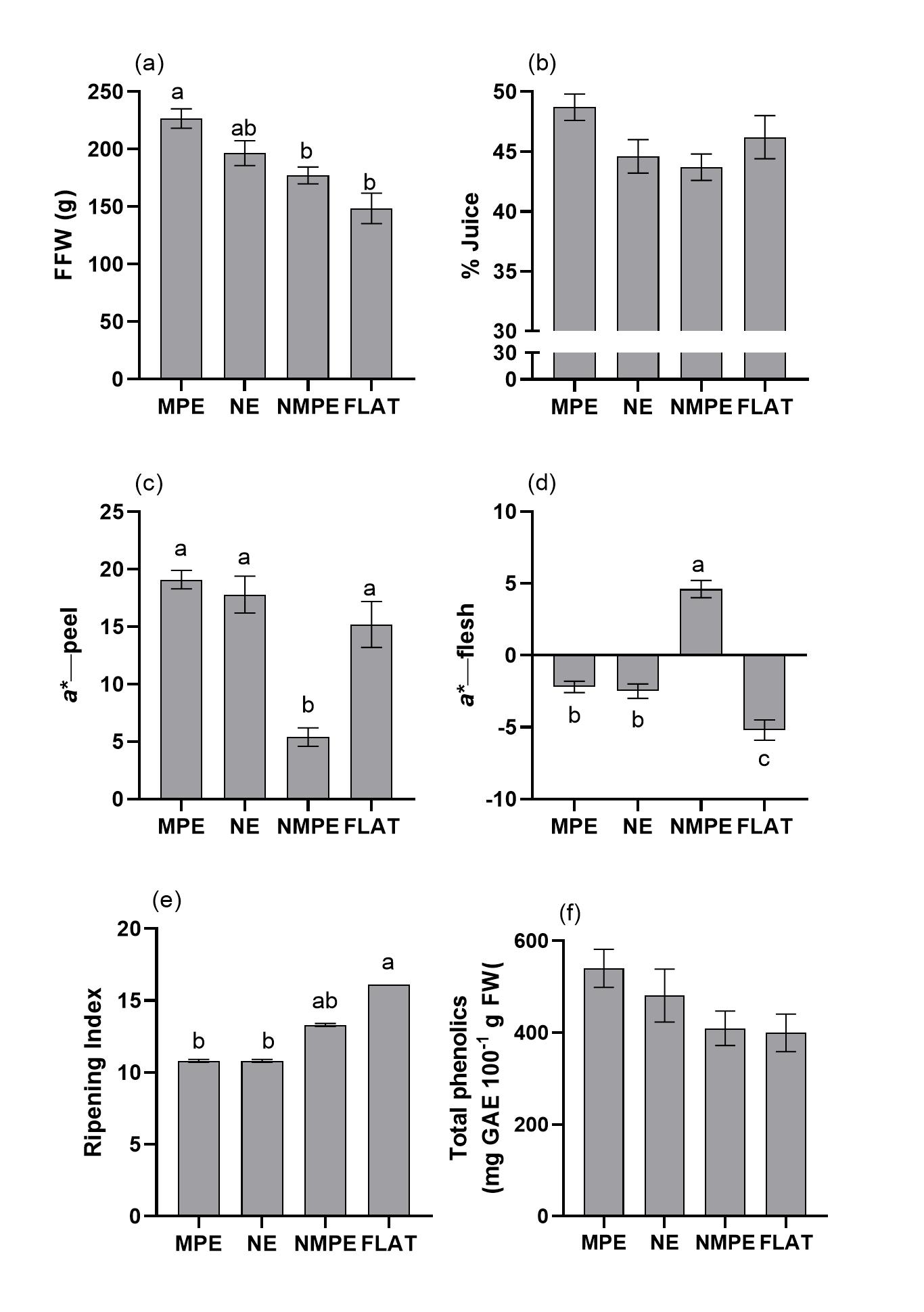

Supplement: Supplementary file 1 — Figure S1. Mean values (±SE) for the fruit type effects on various physical and chemical traits in 100 peach cultivars. Means with different letters indicate difference among separate treatment means. [file JSFA-106-6050-s003.jpg]

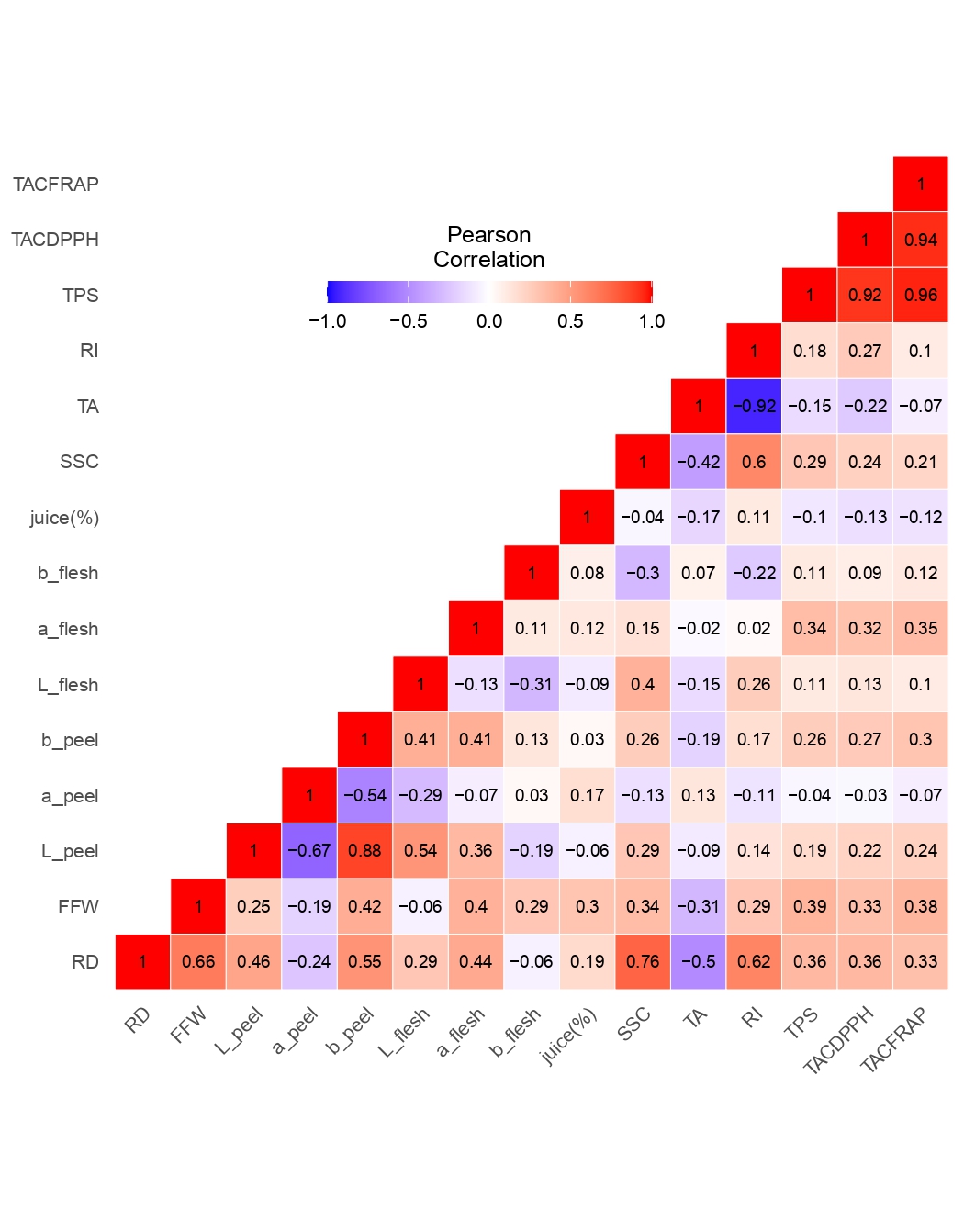

Supplement: Supplementary file 2 — Figure S2. Heat map of Pearson correlation (r) coefficient matrix between fruit phenotyping traits, in 50 melting peach cultivars. Correlations significant differences: *: P < 0.05, **: P < 0.01, ***: P < 0.001. [file JSFA-106-6050-s002.jpg]

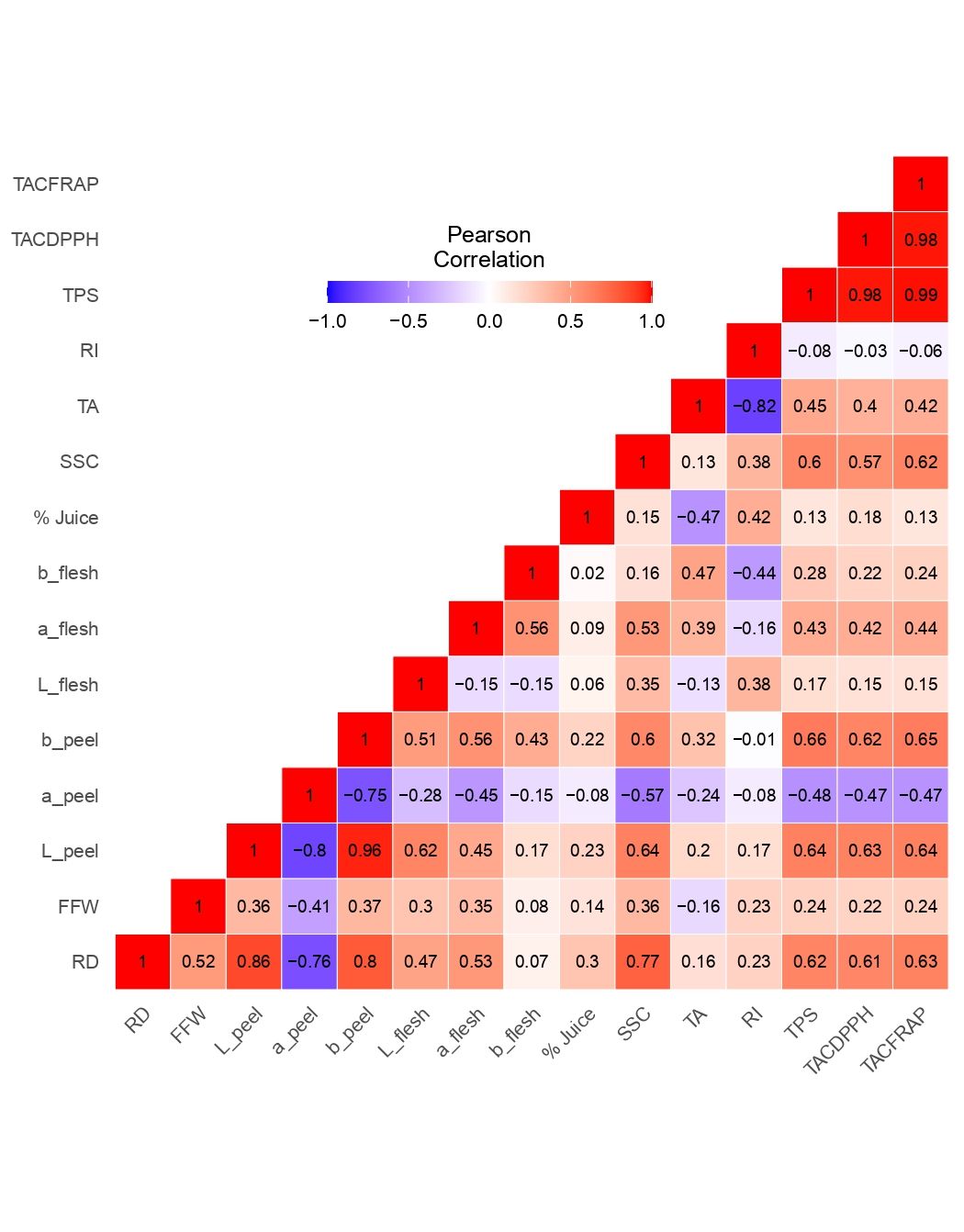

Supplement: Supplementary file 3 — Figure S3. Heat map of Pearson correlation (r) coefficient matrix between fruit phenotyping traits, in 25 nectarine cultivars. Correlations significant differences: *: P < 0.05, **: P < 0.01, ***: P < 0.001. [file JSFA-106-6050-s005.jpg]

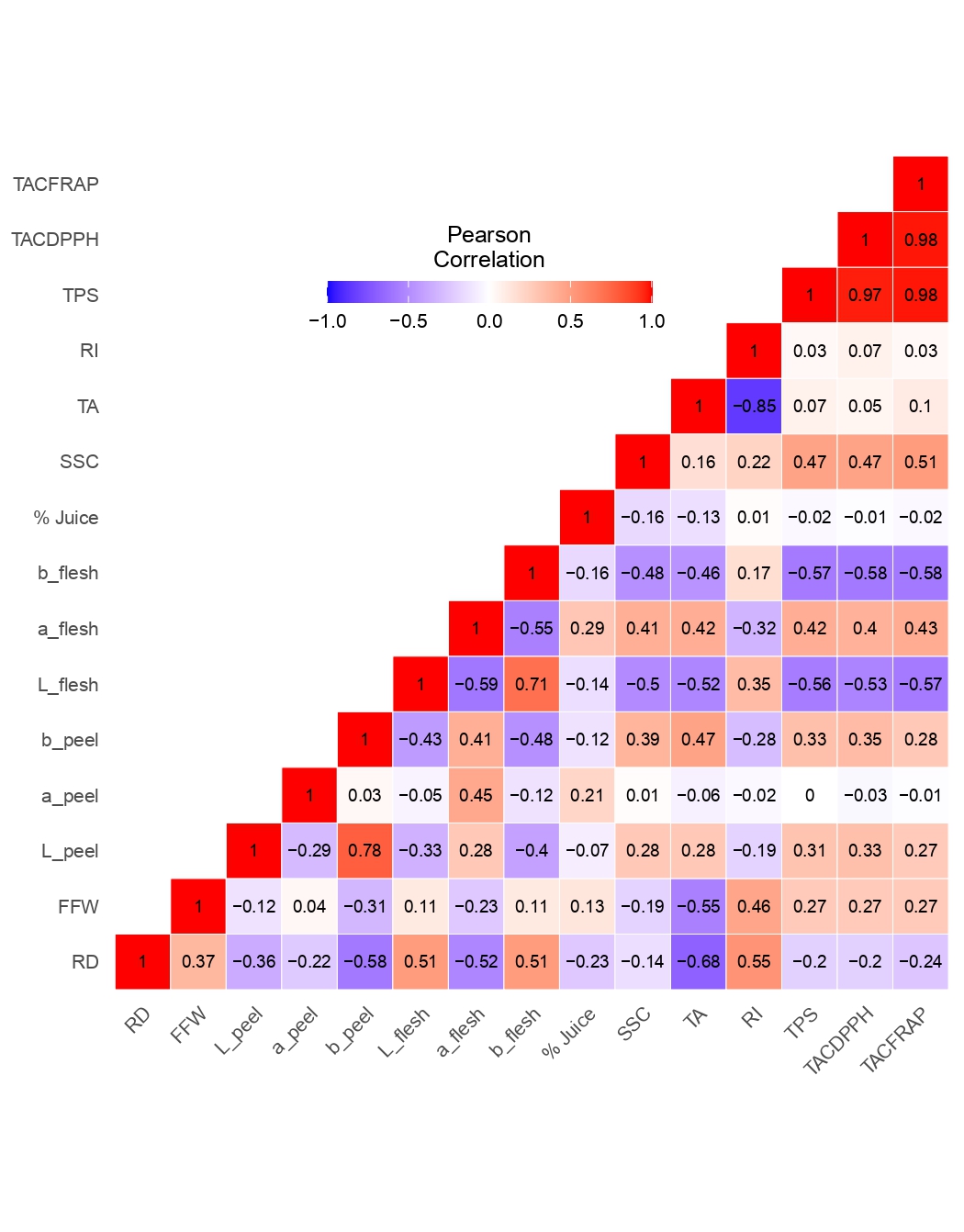

Supplement: Supplementary file 4 — Figure S4. Heat map of Pearson correlation (r) coefficient matrix between fruit phenotyping traits, in 25 nonmelting peach cultivars. Correlations significant differences: *: P < 0.05, **: P < 0.01, ***: P < 0.001. [file JSFA-106-6050-s001.jpg]

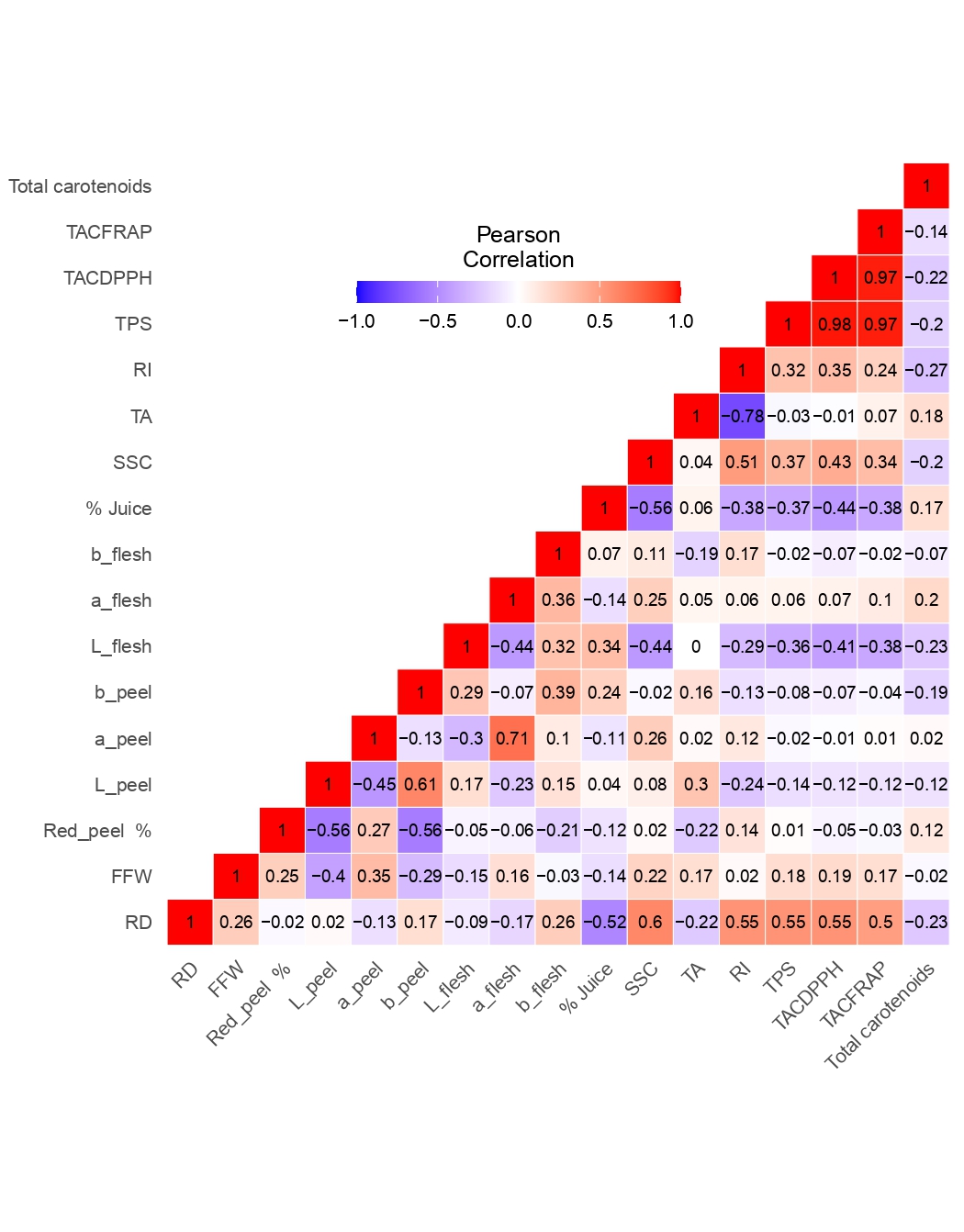

Supplement: Supplementary file 5 — Figure S5. Heat map of Pearson correlation (r) coefficient matrix between fruit phenotyping traits, in 32 apricot cultivars. Correlations significant differences: *: P < 0.05, **: P < 0.01, ***: P < 0.001. [file JSFA-106-6050-s004.jpg]
